# Statistical Surveillance of Structural Breaks in Credit Rating Dynamics

**DOI:** 10.3390/e22101072

**Published:** 2020-09-24

**Authors:** Haipeng Xing, Ke Wang, Zhi Li, Ying Chen

**Affiliations:** 1 Department of Applied Mathematics and Statistics, State University of New York, Stony Brook, NY 11794, USA; zhi.li.3@stonybrook.edu; 2 Quantitative Research, J.P. Morgan Chase, 383 Madison Ave, NY, 10179, USA; wkkate6@gmail.com; 3 Point72 Asset Management, 55 Hudson Yards, New York, NY 10001, USA; yingemma.chen@gmail.com

**Keywords:** credit rating, financial surveillance, rating transition dynamics, statistical process control, structural break

## Abstract

The 2007–2008 financial crisis had severe consequences on the global economy and an intriguing question related to the crisis is whether structural breaks in the credit market can be detected. To address this issue, we chose firms’ credit rating transition dynamics as a proxy of the credit market and discuss how statistical process control tools can be used to surveil structural breaks in firms’ rating transition dynamics. After reviewing some commonly used Markovian models for firms’ rating transition dynamics, we present several surveillance rules for detecting changes in generators of firms’ rating migration matrices, including the likelihood ratio rule, the generalized likelihood ratio rule, the extended Shiryaev’s detection rule, and a Bayesian detection rule for piecewise homogeneous Markovian models. The effectiveness of these rules was analyzed on the basis of Monte Carlo simulations. We also provide a real example that used the surveillance rules to analyze and detect structural breaks in the monthly credit rating migration of U.S. firms from January 1986 to February 2017.

## 1. Introduction

The 2007–2008 global financial crisis, originally triggered by the U.S. subprime mortgage crisis in 2007 and quickly spread over the U.S. and the rest of the world via the U.S. and international banking systems, caused severe economic, political, and social consequences over the world. The crisis not only triggered the European Debt Crisis that began in the European Union at the end of 2009, but also led to a global economic recession which was considered the most severe one since the Great Depression in 1930s. In addition to this, the COVID-19 pandemic declared by the World Health Organization on March 2020 placed the global financial system under further strain and triggered a global economic downturn [1]. These events raise some important questions for economists, financial practitioners, and government regulators, and one of them is whether quantitative tools can be developed to surveil the occurrence of crises or structural breaks in the credit market or its sub-markets.

The question is difficult to answer directly because, in contrast to most quickest change detection problems in which the system dynamics are known, dynamical models have not been found which can extract and aggregate credit information at the microeconomic or macroeconomic level, and in the meanwhile, describe the general movement of the credit market over time. Hence, in order to provide possible insights into the question of detecting structural changes in the credit market, this paper proposes to use firms’ rating transition dynamics as a proxy of the credit market and presents several surveillance rules for structural breaks in firms’ credit rating dynamics.

The reason for choosing firms’ credit ratings as a proxy of the credit market is the following. First, as an opinion on the relative ability of an entity to meet a financial commitment provided by credit rating agencies (CRAs), credit ratings are an information good at the intersection between the demand and supply for capital, which mitigates the information asymmetry in the credit market and hence enhances capital market efficiency and transparency [2]. Second, besides several types of information revealed by firms’ credit rating transitions, such as the conflicts of interest between investors and CRAs [3,4], the effect of a CRA’s reputational concern on rating quality [5,6], the interaction between the business cycle and firms’ incentives [7], and so forth, it has been shown that another type of information, structural breaks in the credit market, can be extracted from firms’ credit rating transitions [8,9,10]. Third, credit rating models play an important role in modern credit risk management. For example, in the pricing of bonds and credit, derivative, structural, and reduced-form models are used to model firms’ credit risk and credit ratings [10,11,12,13,14]; in bank regulation, banks are required to construct credit rating models to stress test their portfolios and evaluate evidence of rating transitions in external ratings [15,16].

By choosing firms’ rating transition dynamics as a proxy of the credit market, this paper makes the following contributions to the literature of financial surveillance. First, to the best of our knowledge, this is the first study that extends statistical process control methods to study the problem of monitoring financial stability at the regulatory level. Specifically, we combine rules of change-point detection with unknown pre- and after-change distributions with the state-of-the-art models of credit rating dynamics and propose different types of surveillance rules for structural breaks in rating transition dynamics. Second, instead of using a single or aggregated time series that is summarized from the credit market, the surveillance methods proposed in the paper are based on credit behavior of a collection of firms, which not only provides each firm’s credit quality at the microeconomic level but also aggregates the structural break information over firms for the surveillance purpose. Third, the empirical study in the paper compares the proposed surveillance rules for structural breaks in firms’ rating transition dynamics and demonstrates the effectiveness of these rules in the surveillance of the credit market.

We next elaborate the proposed methods and empirical study as follows. Since firms’ rating transition dynamics have been chosen as a proxy of the credit market, one can link the variability of rating transition dynamics with the stability of the credit market. Before presenting surveillance rules for firms’ credit rating dynamics, we review models that characterize firms’ credit rating migrations as discrete- or continuous-time Markov chains in Section 2. A typical model of these assumes that firms’ rating migrations follow discrete-time homogeneous Markov chain, which is characterized by a probability transition matrix. Though it is usually used by CRAs to publish firms’ yearly rating transition matrices, the discrete-time model is criticized due to the availability of rating data and the need for forecasting rating transition matrices over any time horizon. Therefore, firms’ rating transitions are also modeled in continuous time and all surveillance rules discussed in this paper are also based on continuous-time settings. We consider two types of continuous-time models in the paper. The first one assumes that firms’ rating transitions follow continuous-time homogeneous Markov chains that are characterized by a generator matrix of probability transition matrices. The second model is the stochastic structural break (SSB) [8], which assumes that firms’ rating transitions follow a continuous-time piecewise homogeneous Markov chain characterized by piecewise constant generator matrices. The SSB model assumes the existence of multiple unknown structural breaks in firms’ rating transition dynamics and provides attractive online estimates of generator matrices and probabilities of structural breaks.

We then present several surveillance rules based on continuous-time models of firms’ rating transition dynamics. Since both the pre- and after-change distributions of firms’ rating generator matrices are unknown, rules of detecting structural breaks in firms’ rating dynamics involve steps of estimating pre- and after-change distributions. In particular, we consider the following rules. The first rule is based on the likelihood ratio tests using historical and current rating records, which can be further specified as maximized likelihood ratios and mixture likelihood ratios. The second rule involves the generalized likelihood ratio test when firms’ rating transitions follow continuous-time Markov chains with different generators before and after the break point. The third rule applies the extended Shiryaev’s detection rule [17] to continuous-time Markov chains with different pre- and after-change generators. The fourth rule is a Bayesian detection rule for exponential families with unknown multiple change-points [18] and is based on estimated probabilities of the most recent change-point. We apply the fourth rule to the SSB model for firms’ rating transition dynamics and obtain a surveillance rule for structural breaks.

As general properties of these surveillance rules have been discussed in the literature, we focus on the effectiveness of these rules for detecting changes in firms’ rating transition dynamics. Section 4 describes our simulation studies for detecting structural breaks in four-state continuous-time Markov chains with break points in generator matrices. In particular, we discuss the false alarm probabilities and conditional detection delay of the four surveillance rules in different scenarios. To further demonstrate the performance of these surveillance rules in detecting structural breaks in firms’ rating transition dynamics, Section 5 walks through a real example in which structural breaks in the U.S. firms’ monthly rating transitions are detected. In particular, after a preliminary analysis of the U.S. firms’ rating transitions during 1986–2016, we applied the surveillance rules discussed in Section 3 and Section 4 and detect sequentially structural breaks in firms’ rating transition dynamics from January 1997 to February 2017.

The result in Section 5 shows that surveillance rules can help us detect structural breaks in firms’ rating transition dynamics, and some of detected break points can be traced back to economic events that might cause structural changes of the credit market. However, structural breaks detected by different surveillance rules can be very different, indicating that further studies need to be carried out. Section 6 concludes the paper with some remarks.

## 2. Models of Credit Rating Dynamics

In the study of credit rating dynamics, it is convenient to assume that a firm’s credit ratings follow a time-homogeneous Markov chain. Suppose that there are *K* rating categories which are ordered as {1,⋯,K} from excellent to very bad credit quality. In particular, we denote category *K* a “default” state of the firm which is an absorbing state.

### 2.1. Discrete-Time Homogeneous Rating Transition Matrices

Consider first a discrete-time setting for firms’ rating transitions. Denote by $\eta_{t}\in \left\{1,\cdots ,K\;\right\}$ the rating of firm *l* at time *t*. Suppose that ${\left\{\eta_{t}\right\}}_{t\geq 0}$ follows a discrete-time *K*-state Markov chain, which is characterized by a probability transition matrix $P={\left(p_{ij}\right)}_{1\leq i,j\;\leq K}$ and $p_{ij}=P\left(\eta_{t}=j|\eta_{t-1}=i\right)$ is the probability that firm *l* migrates from rating category *i* at time t−1 to rating category *j* at time *t*. Then for i=1, ⋯,K, $\sum_{j=1}^{K}p_{ij}=1$, and $p_{KK}=1$ since *K* is a default state. The probability transition matrix P is a one-period (usually, one-year) transition matrix, for rating transitions over multiple periods; the transition probability matrix can be obtained by matrix multiplication. In particular, the firm *l*’s (t−s)-period (t>s≥0) probability transition matrix is expressed as $P^{t-s}$.

Suppose that all firms’ rating transitions follow a discrete-time *K*-state Markov chain with one-year probability transition matrix $P={\left(p_{ij}\right)}_{1\leq i,j\;\leq K}$. Assume that there are $N_{i}$ firms in a given rating category *i* at the time t−1 and $N_{ij}$ firms migrating from *i* at time t−1 to *j*(j≠i) at time *t*; the log-likelihood of observed ratings from t−1 to *t* is

(1) $$\log L\left(P|Y_{t-1,t}\right)=\sum_{i=1}^{K}\left[\sum_{j\neq i}N_{ij}\log p_{ij}+\left(N_{i}-\sum_{j\neq i}N_{ij}\right)\log\left(1-\sum_{j\neq i}p_{ij}\right)\right].$$

Then the maximum likelihood estimate for P is given by ${\hat{p}}_{ij}=N_{ij}/N_{i}$ for j≠i and ${\hat{p}}_{ii}=1-\sum_{j\neq i}{\hat{p}}_{ij}$ for i=1,⋯,K.

In the literature of measuring changes of probability transition matrices for general Markov chains, mobility indices of probability transition matrices are usually used. Ref. [19] proposed indices for Markov matrices using eigenvalues and determinants. Ref. [20] presented a set of criteria by which the performance of a proposed metric for arbitrary transition matrices should be judged. In particular, [20] summarized criteria in three distinct aspects. The persistence criteria require that a metric is consistent with some simple, intuitively appealing interpretations of the transition matrix P; the convergence criteria specify that a metric needs to establish an ordering among transition matrices P that is consistent with the rate at which the multiperiod transition matrices $P^{s}$(s>0 is an integer) converge to a limiting transition matrix; and the temporal aggregation criteria remove the influence of the length of the basic time period on comparisons of mobility. Ref. [21] argued that, since credit migration matrices are generally diagonally dominant and then the decay rates towards its steady-state are generally slow, the temporal aggregation criteria can be neglected given that credit migration matrices are usually evaluated for fixed periods considerably shorter than the decay time of the system. Instead, [21] highlighted the importance of an additional persistence, distribution discriminatory criterion, which can discriminate matrices with the same row-wise probabilities of change but different distributions across each row, and then proposed a scalar SVD metric defined as the average of the singular values of the mobility matrix for a credit migration matrix P:

(2) $$M\left(P\right)=\frac{1}{K}\sum_{i=1}^{K}\sqrt{e_{i}\left[{\left(P-I\right)}^{\prime }\left(P-I\right)\right]},$$

in which I is a K×K identity matrix and $e_{i}\left(\cdot \right)$ is the *i*th eigenvalue of the matrix. Note that P−I represents the magnitude of the implied mobility of migration matrix. Ref. [8] later used the SVD metric to show time variations in estimated credit migration matrices.

One concern regarding the discrete-time method of a estimating credit rating transition matrix is that the estimated probability of rare events can be zero due to the the observability of the rare events. Besides, the method does not provide transition probabilities in fractional periods ([22], Chapter 6). Since the continuous-time history of rating transitions is usually accessed by the rating agencies or banks using an internal rating system, a continuous-time homogeneous Markov framework is usually assumed for the rating process.

### 2.2. Continuous-Time Homogeneous Markov Transition Matrices

Suppose that the rating migration process of a firm for the period (0,t) is a continuous-time homogeneous Markov chain with transition matrix P(0,t), in which the ijth entry represents the probability of migrating from category *i* to category *j* during the period (0,t). Similarly to the discrete-time Markov process for which the rating transition matrix can be obtained by matrix multiplication from the one-period transition matrix, the matrix P(0,t) can be obtained through its generator matrix Λ under the assumption of time homogeneity. That is, for any time t>0,

(3) $$P\left(0,t\right)=\exp\left(\Lambda t\right):=\sum_{k=0}^{\infty }\frac{\Lambda^{k}t^{k}}{k!},$$

in which $\Lambda =(\lambda^{\left(i,j\right)})$ satisfies $\lambda^{\left(i,i\right)}=-\sum_{j\neq i}\lambda^{\left(i,j\right)}$ for 1≤i≤K, and $\lambda^{\left(i,j\right)}\geq 0$ for 1≤i≠j≤K. Note that the elements of the last row in the generator matrix Λ represent the rating transitions from the default category to others and are usually considered as zeros.

Suppose that there are *n* realizations of a Markov chain with the generator matrix Λ. Denote by, for the period (0,t), $K_{0,t}^{\left(i,j\right)}$ the number of transitions from category *i* to category *j*, $S_{0,t}^{\left(i\right)}$ the amount of time spent in category *i*, and $Y_{0,t}$ the observed rating transitions over the period (0,t). The log-likelihood of $Y_{0,t}$ given the generator Λ is expressed as

(4) $$\log L\left(\Lambda |Y_{0,t}\right)=\sum_{i=1}^{K}\left[\sum_{j\neq i}K_{0,t}^{\left(i,j\right)}\log\lambda^{\left(i,j\right)}-\left(\sum_{j\neq i}\lambda^{\left(i,j\right)}+1-K\right)S_{0,t}^{\left(i\right)}\right];$$

see [23]. Then the maximum likelihood estimates of elements of Λ are given by ${\hat{\lambda }}^{\left(i,j\right)}=K_{0,t}^{\left(i,j\right)}/S_{0,t}^{\left(i\right)}$.

One may also assume a prior distribution for the off-diagonal elements $\lambda^{\left(i,j\right)}$ and obtain a Bayesian estimate of the generator matrix. In particular, suppose that the off-diagonal elements $\lambda^{\left(i,j\right)}$ follow independently a gamma$\left(\alpha_{ij},\beta_{i}\right)$ prior distribution with the density function

(5) $$g\left(\lambda^{\left(i,j\right)}\right)=\frac{\beta_{i}^{\alpha_{ij}}}{\Gamma (\alpha_{ij})}{\left[\lambda^{\left(i,j\right)}\right]}^{\alpha_{ij}-1}\exp\left(-\lambda^{\left(i,j\right)}\beta_{i}\right),\;\left(i,j\right)\in K,$$

in which K={(i,j)|i≠j,1≤i≤K−1,1≤j≤K}. Combining the conjugate gamma prior (5) with the likelihood function (4) yields the posterior distribution of $\lambda^{\left(i,j\right)}$ given $Y_{0,t}$, which is gamma$\left(K_{0,t}^{\left(i,j\right)}+\alpha_{ij},S_{0,t}^{\left(i\right)}+\beta_{i}\right)$. Then the element $\lambda^{\left(i,j\right)}$ can be estimated by the posterior mean of the gamma distribution, i.e., ${\hat{\lambda }}^{\left(i,j\right)}=\left(K_{0,t}^{\left(i,j\right)}+\alpha_{ij}\right)/\left(S_{0,t}^{\left(i\right)}+\beta_{i}\right)$.

### 2.3. Continuous-Time Piecewise-Homogeneous Transition Matrices

Some empirical studies show that the observed firms’ rating transitions are not stationary over time, so we may move forward and assume that a firm’s rating transition process follows a *K*-state non-homogeneous continuous time Markov process. In general, such a process can be characterized by a transition probability matrix P(s,t) over the period (s,t), in which the ijth element of P(s,t) represents the probability that a firm starting in state *i* at time *s* is in state *j* at time *t*. Suppose that there are *m* rating transitions observed over the period (s,t). For a transition time $t_{k}$ in (s,t), denote by $\Delta N_{ij}\left(t_{k}\right)$ the number of transitions observed from state *i* to state *j* at time $t_{k}$, $\Delta N_{i}\left(t_{k}\right)=\sum_{1\leq j\leq K,j\;\neq i}\Delta N_{ij}\left(t_{k}\right)$, and $Y_{i}\left(t_{k}\right)$ the number of firms in state *i* right before time $t_{k}$. The non-homogeneous transition matrix P(s,t) can be consistently estimated by the product-limit estimator

(6) $$\hat{P}\left(s,t\right)=\prod_{k=1}^{m}\left(I+\Delta \hat{A}\left(t_{k}\right)\right),$$

in which

$$\Delta \hat{A}\left(t_{k}\right)=\left(\begin{array}{ccccc} -\frac{\Delta N_{1}\left(t_{k}\right)}{Y_{1}\left(t_{k}\right)} & \frac{\Delta N_{12}\left(t_{k}\right)}{Y_{1}\left(t_{k}\right)} & \frac{\Delta N_{13}\left(t_{k}\right)}{Y_{1}\left(t_{k}\right)} & \cdots & \frac{\Delta N_{1K}\left(t_{k}\right)}{Y_{1}\left(t_{k}\right)}\\ \frac{\Delta N_{21}\left(t_{k}\right)}{Y_{2}\left(t_{k}\right)} & -\frac{\Delta N_{2}\left(t_{k}\right)}{Y_{2}\left(t_{k}\right)} & \frac{\Delta N_{23}\left(t_{k}\right)}{Y_{2}\left(t_{k}\right)} & \cdots & \frac{\Delta N_{2K}\left(t_{k}\right)}{Y_{2}\left(t_{k}\right)}\\ \vdots & \vdots & & \cdots & \vdots \\ \frac{\Delta N_{K-1,1}\left(t_{k}\right)}{Y_{K-1}\left(t_{k}\right)} & \frac{\Delta N_{K-1,2}\left(t_{k}\right)}{Y_{K-1}\left(t_{k}\right)} & \cdots & -\frac{\Delta N_{K-1}\left(t_{k}\right)}{Y_{K-1}\left(t_{k}\right)} & \frac{\Delta N_{K-1,K}\left(t_{k}\right)}{Y_{K-1}\left(t_{k}\right)}\\ 0 & 0 & \cdots & \cdots & 0 \end{array}\right);$$

see [24]. In the matrix above, the *k*th diagonal element counts the fraction of the exposed firms $Y_{i}\left(t_{k}\right)$ leaving the state at time $t_{k}$, and the (ij)’th off-diagonal element counts the fraction of transitions from the category *i* to the category *j* in the number of exposed firms at time $t_{k}$. Note that the variable *Y* has incorporated the case of censoring for which there is no change in the estimator at the time of a censoring event. Furthermore, the last row in $\Delta \hat{A}\left(t_{k}\right)$ is zero because the *K*th state (i.e., default state) is absorbent.

The non-homogeneous transition matrix P(s,t) and its product-limit estimator (6) do not have any specification for the time-homogeneity of rating migrations, and hence cannot directly be used to model the dynamics of rating migrations. To overcome this difficulty, the SSB model in [8] assumed that the non-homogeneous Markov transitions of firms’ ratings can be decomposed as piecewise homogeneous continuous-time Markov processes. There are two key assumptions in the stochastic structural break model. The first assumption is that structural breaks in the time-varying generator matrices Λ(t) follow a Poisson process $\left\{N_{\Lambda }\left(t\right);t\geq 0\right\}$ with constant rate η and generator matrices between two consecutive structural breaks are constant. Then given times of structural breaks $\tau_{1},\cdots ,\tau_{M}$ in the period (s,t), the transition matrix in the period (s,t) can be characterized as

(7) $$P\left(s,t\right)=\prod_{k=1}^{M+1}P\left(\tau_{k-1},\tau_{k}\right)=\prod_{k=1}^{M+1}\exp\left(\int_{\tau_{k-1}}^{\tau_{k}}\Lambda \left(u\right)du\right)=\prod_{k=1}^{M+1}\exp\left[\left(\tau_{k}-\tau_{k-1}\right)\Lambda \left(\tau_{k}-\right)\right],$$

in which $\tau_{0}=s,\tau_{M+1}=t$. Note that matrices $P\left(\tau_{0},\tau_{1}\right),\cdots ,P\left(\tau_{M},\tau_{M+1}\right)$ in (7) are generally not commutable. In the special case that there are no break times in the period (s,t), P(s,t) becomes homogeneous and is expressed as

$$P\left(s,t\right)=\exp\left(\int_{s}^{t}\Lambda \left(u\right)du\right)=\exp\left[(t-s)\Lambda (t-)\right].$$

The second assumption of the model is that the piecewise constant generator matrices $\Lambda (\tau_{k})$ are independent and identically distributed (i.i.d.) random matrices and their off-diagonal element $\lambda^{\left(i,j\right)}\left(\tau_{k}\right)$ follows a gamma$\left(\alpha_{ij},\beta_{i}\right)$ prior distribution whose density function is given by (5).

For the period (s,t), denote by $K_{s,t}^{\left(i,j\right)}$ the number of transitions from category *i* to category *j*, $S_{s,t}^{\left(i\right)}$g the amount of time spent in category *i*, $\lambda_{s,t}^{\left(i,j\right)}$ the ijth entry in the generator Λ(t), and $Y_{s,t}$ the observed rating transitions over the period (s,t). The SSB model suggests a filtering approach to estimate the piecewise constant generator matrices Λ(t). In particular, they partitioned the sample period (0,T) as $0=t_{0}<t_{1}<\cdots <t_{L}=T$ and assumed that structural breaks can only occur at the times $t_{1},\cdots ,t_{L}$. Let $I_{1}=1$ and $I_{l}=N_{\Lambda }\left(t_{l}-\right)-N_{\Lambda }\left(t_{l-1}-\right)$ for l=2,⋯,L; then $I_{l}$ indicates if Λ(t) are the same during the periods $\left(t_{l-2},t_{l-1}\right)$ and $\left(t_{l-1},t_{l}\right)$ and $\left\{I_{l}\right\}$ are a sequence of i.i.d. Bernoulli random variables with success probability p=1−exp(−ηT/L). To derive the posterior distribution of $\Lambda \left(t_{l}\right)={\left(\lambda_{t_{l-1},t_{l}}^{\left(i,j\right)}\right)}_{(i,j)\in K}$ given $Y_{\left(0,T\right)}$ they further computed the probability that the most recent break time prior to $t_{l-1}$ is $t_{m-1}$, i.e., $p_{m,l}=P\left(R_{l}=t_{m-1}|Y_{t_{m-1},t_{l}}\right)$, where $R_{l}=\max\left\{t_{m-1}|I_{m}=1,m\leq l\right\}$ is the most recent break time up to time $t_{l-1}$, and showed that the posterior distribution of $\lambda_{t_{l-1},t_{l}}^{\left(i,j\right)}$ given $Y_{\left(0,t_{l}\right)}$ is a mixture of gamma distributions,

(8) $$\lambda_{t_{l-1},t_{l}}^{\left(i,j\right)}|Y_{\left(0,t_{l}\right)}∼\sum_{m=1}^{l}p_{m,l}Gamma\left(K_{t_{m-1},t_{l}}^{\left(i,j\right)}+\alpha_{ij},S_{t_{m-1},t_{l}}^{\left(i\right)}+\beta_{i}\right).$$

The mixture weight can be calculated recursively by $p_{m,l}=p_{m,l}^{*}/\sum_{m=1}^{l}p_{m,l}^{*}$, in which

(9) $$p_{m,l}^{*}=\left\{\begin{array}{cc} pf_{l,l}/f_{0,0} & m=l,\\ \left(1-p\right)p_{m,l-1}f_{m,l}/f_{m,l-1} & m<l, \end{array}\right.$$

and the terms $f_{m,l}$ and $f_{0,0}$ are expressed as

(10) $$f_{m,l}=\prod_{i,j\in K}\Gamma \left(K_{t_{m-1},t_{l}}^{\left(i,j\right)}+\alpha_{ij}\right)/{\left(S_{t_{m-1},t_{l}}^{\left(i\right)}+\beta_{i}\right)}^{\left(K_{t_{m-1},t_{l}}^{\left(i,j\right)}+\alpha_{ij}\right)},\;f_{0,0}=\prod_{i,j\;\in K}\Gamma \left(\alpha_{ij}\right)/\beta_{i}^{\alpha_{i,j}}.$$

Xing et al. (2012) shows that, provided the above forward filter for generator matrices, the backward filter can be similarly obtained and then smoothing estimates of $\lambda_{t_{l-1},t_{l}}^{\left(i,j\right)}$ given all the data $Y_{\left(0,T\right)}$ can be derived.

The forward estimates of generator matrices (8) also provide a statistic related to structural breaks, which is the probability that the most recent change-point occurs in the period $\left[t_{h-1},t_{l}\right]$ up to time $t_{l}$:

(11) $$P\left(R_{h}\in \left[t_{h-1},t_{l}\right]|Y_{0,t_{l}}\right)=\sum_{h=m}^{l}p_{m,l}.$$

We use (11) to construct a surveillance rule for structural breaks in the next section.

## 3. Methods for Surveillance

We now consider several surveillance rules for structural breaks in the generator of rating migration matrices, using the continuous-time models of rating transition dynamics in Section 2.2 and Section 2.3. Suppose that firms’ rating migrations follow a *K*-state continuous-time Markov chain whose probability transition matrices are characterized by their generator matrices Λ(t). Denote by $Y_{s,t}$ the observed ratings during the period (s,t); then its sufficient statistic is given by $\left\{K_{s,t}^{\left(i,j\right)},S_{s,t}^{\left(i\right)}\;|\;1\leq i,j\leq K\right\}$, where $K_{s,t}^{\left(i,j\right)}$ and $S_{s,t}^{\left(i\right)}$ are defined in Section 2.3. Assume that the generator matrix Λ(t) is $\Lambda_{0}$ for t<ν and $\Lambda_{1}$ for t≥ν.

If both $\Lambda_{0}$ and $\Lambda_{1}$ are known, one may consider the cumulative sum (CUSUM) rule [25] to detect the change-point ν,

(12) $$N_{\mathrm{CUSUM}}=\inf\left\{n:\max_{0\leq k<n}\log\left[L\left(\Lambda_{1};Y_{k+1,n}\right)/f\left(\Lambda_{0};Y_{k+1,n}\right)\right]\geq c\right\}$$

with *c* satisfying $E_{0}\left(N_{\mathrm{CUSUM}}\right)=\gamma$. Ref. [26] showed the asymptotic minimax property of the CUSUM rule, and it suggests that as γ→∞, the CUSUM rule (12) asymptotically minimizes the worst-case expected delay ${\bar{E}}_{1}\left(T\right)=\sup_{v\geq 1}\mathrm{ess}\sup E\left[{\left(T-v+1\right)}^{+}|Y_{0,\nu }\right]$ over the class $F_{\gamma }$ of all rules *T* satisfying the constraint $E_{0}\left(T\right)\geq \gamma$, and furthermore, as γ→∞, ${\bar{E}}_{1}\left(N_{\mathrm{CUSUM}}\right)∼\inf_{T\in F_{\gamma }}{\bar{E}}_{1}\left(T\right)∼\left(\log\gamma \right)/I\left(L_{1},L_{0}\right)$, where $I\left(L_{1},L_{0}\right)=E_{1}\{\log\left(L\left(\Lambda_{1}|Y_{t-1,t}\right)/L\left(\Lambda_{0}|Y_{t-1,t}\right)\right\}$ is the Kullback–Leiber information number.

The CUSUM rule cannot be used to detect change-points (or structural breaks) in rating transition dynamics since the pre- and after-changes of generator matrices are unknown by definition of the problem. Hence we consider the following surveillance rules that assume either $\Lambda_{1}$ is unknown or both $\Lambda_{0}$ and $\Lambda_{1}$ are unknown. To better present the idea, we ignore the assumption that state *K* is an absorbing state in this section and will make necessary modifications on discussed rules later for real data implementation.

### 3.1. Likelihood Ratio (LR) Tests

To study sequentially whether there is a change in the generator matrices of firms’ rating transition process, we first consider a hypothesis testing problem for observed ratings transitions $Y_{t-1,t}$ at the period (t−1,t], $H_{0}:\Lambda =\Lambda_{0}$ versus $H_{1}:\Lambda \neq \Lambda_{0}$, where the generator matrix $\Lambda_{0}={\left(\lambda_{0}\right)}_{1\leq i,j\;\leq K}$.

Since the log-likelihood function of the data for generator matrix Λ is given by (4), the logarithm of the likelihood ratio statistic for the hypothesis testing problem is

(13) $$\begin{array}{cc} \; & Z_{\mathrm{LR}}\left(\Lambda_{0};Y_{t-1,t}\right):=2\left(\max_{\Lambda \neq \Lambda_{0}}\log L\left(\Lambda |Y_{t-1,t}\right)-\log L\left(\Lambda_{0}|Y_{t-1,t}\right)\right)\\ = & 2\sum_{i=1}^{K}\left[\sum_{j\neq i}K_{t-1,t}^{\left(i,j\right)}\log\frac{{\hat{\lambda }}^{\left(i,j\right)}}{\lambda_{0}^{\left(i,j\right)}}-\sum_{j\neq i}\left({\hat{\lambda }}^{\left(i,j\right)}-\lambda_{0}^{\left(i,j\right)}\right)S_{t-1,t}^{\left(i\right)}\right], \end{array}$$

where ${\hat{\lambda }}^{\left(i,j\right)}=K_{t-1,t}^{\left(i,j\right)}/S_{t-1,t}^{\left(i\right)}$ is the maximum likelihood estimate (MLE) of $\lambda^{\left(i,j\right)}$(1≤i≠j≤K). Besides using the MLE of Λ, one may specify gamma prior distributions for elements of Λ and obtain a mixture likelihood ratio (MLR). Suppose elements of the generator matrix Λ follow the gamma distribution (5); the MLR of the data is expressed as

(14) $$\begin{array}{cc} \; & Z_{\mathrm{MLR}}\left(\Lambda_{0};Y_{t-1,t}\right):=2\log\left(\int L\left(\Lambda |Y_{t-1,t}\right)g\left(\Lambda \right)d\Lambda \right)-2\log L\left(\Lambda_{0}|Y_{t-1,t}\right)\\ = & 2\sum_{i=1}^{K}\{\sum_{j\neq i}[\alpha_{ij}\log\beta_{i}-\log\Gamma \left(\alpha_{ij}\right)-\left(K_{t-1,t}^{\left(i,j\right)}+\alpha_{ij}\right)\log\left(S_{t-1,t}^{\left(i\right)}+\beta_{i}\right)\\ \; & \;+\log\Gamma \left(K_{t-1,t}^{\left(i,j\right)}+\alpha_{ij}\right)-K_{t-1,t}^{\left(i,j\right)}\log\lambda_{0}^{\left(i,j\right)}]+\left(\sum_{j\neq i}\lambda_{0}^{\left(i,j\right)}+1-K\right)S_{t-1,t}^{\left(i\right)}\}. \end{array}$$

Based on the LR and MLR statistics, (13) and (14), we obtain the following rules that detect whether a change-point occurs at the period $Y_{t,t+1}$ in the generator of rating migration matrices,

(15) $$N_{\mathrm{LR}}=\inf\left\{t\geq t_{0}:Z_{\mathrm{LR}}\left(\Lambda_{0};Y_{t-1,t}\right)\geq c\right\},$$

and

(16) $$N_{\mathrm{MLR}}=\inf\left\{t\geq t_{0}:Z_{\mathrm{MLR}}\left(\Lambda_{0};Y_{t-1,t}\right)\geq c\right\}.$$

The above two rules assume the pre-change distribution of firms’ rating transitions is known. Since $\Lambda_{0}$ is unknown in reality, one can replace it by its MLE using historical observations. In particular, given the observed rating transitions $Y_{0,t-1}$ during the period (0,t−1], let ${\hat{\Lambda }}_{0}\left(Y_{0,t-1}\right)$ be the MLE of $\Lambda_{0}$ during the period (0,t−1]. Then rules (15) and (16) can be modified as follows:

(17) $${\hat{N}}_{\mathrm{LR}}=\inf\left\{t\geq t_{0}:Z_{\mathrm{LR}}\left({\hat{\Lambda }}_{0}\left(Y_{0,t-1}\right);Y_{t-1,t}\right)\geq c\right\},$$

(18) $${\hat{N}}_{\mathrm{MLR}}=\inf\left\{t\geq t_{0}:Z_{\mathrm{MLR}}\left({\hat{\Lambda }}_{0}\left(Y_{0,t-1}\right);Y_{t-1,t}\right)\geq c\right\}.$$

Besides constructing detection rules directly, the LR and MLR statistics (13) and (14) can also be used to construct exponentially weighted moving average (EWMA) control charts. Control charts were first introduced by [27,28] to monitor production processes and were later extended to different disciplines. The EWMA control chart, based on the EWMA statistic, was introduced by [29]. Its multivariate version was implemented by [30] and extended by [31] to monitor multivariate time series. The EWMA control chart has been applied in financial surveillance. For example, [32] constructed multivariate EWMA control charts based on the Mahalanobis distance to sequentially detect a change in the parameters of the Cox–Ingersoll–Ross term structure model for interest rates. Using the LR and MLR statistics (13) and (14), one can easily construct the following EWMA control charts for generator matrices of rating transition matrices:

$$W_{t,\mathrm{LR}}=\left(1-\alpha \right)W_{t,\mathrm{LR}}+\alpha Z_{\mathrm{LR}}\left({\hat{\Lambda }}_{0}\left(Y_{0,t-1}\right);Y_{t-1,t}\right),\;t\geq 1,$$

$$W_{t,\mathrm{MLR}}=\left(1-\alpha \right)W_{t,\mathrm{LR}}+\alpha Z_{\mathrm{MLR}}\left({\hat{\Lambda }}_{0}\left(Y_{0,t-1}\right);Y_{t-1,t}\right),\;t\geq 1.$$

As it is usually difficult to find the distributions of the above two EWMA statistics, one may use bootstrap methods to estimate the distribution of the EWMA statistics for real data implementation.

### 3.2. Generalized Likelihood Ratio (GLR) Rule

The LR and MLR statistics (or their modified versions) can be considered as likelihood ratio statistics for the problem of testing whether a change point occurs at t−1 given observations $Y_{0,t}$. In general, since both $\Lambda_{0}$ and $\Lambda_{1}$ are unknown in practice, one may consider the GLR statistic for testing the null hypothesis of no change-point based on $Y_{0,1},\cdots ,Y_{t-1,t}$, versus the alternative hypothesis of a single change-point prior to *t* but not before $t_{0}$:

$$\begin{array}{cc} \; & Z_{\mathrm{GLR}}\left(Y_{0,t}\right):=\max_{n_{0}\leq k\leq n}\left\{\underset{\Lambda_{1}}{\sup}\log L\left(\Lambda_{1};Y_{0,k}\right)+\underset{\Lambda_{2}}{\sup}\log L\left(\Lambda_{2};Y_{k,t}\right)-\underset{\Lambda_{0}}{\sup}\log L\left(\Lambda_{0};Y_{0,t}\right)\right\}\\ = & \max_{n_{0}\leq k\leq n}\sum_{1\leq i\neq j\leq K}\left\{K_{0,k}^{\left(i,j\right)}\log\frac{K_{0,k}^{\left(i,j\right)}}{S_{0,k}^{\left(i\right)}}+K_{k,t}^{\left(i,j\right)}\log\frac{K_{k,t}^{\left(i,j\right)}}{S_{k,t}^{\left(i\right)}}-K_{0,t}^{\left(i,j\right)}\log\frac{K_{0,t}^{\left(i,j\right)}}{S_{0,t}^{\left(i\right)}}\right\} \end{array}$$

where $\sup_{\Lambda_{0}}$ is the maximum likelihood under the null hypothesis, and $\sup_{\Lambda_{1}}$ and $\sup_{\Lambda_{2}}$ are obtained by maximizing the likelihood under the hypothesis of a single change-point occurring at k+1. Then the GLR rule with unknown pre- and post-change parameters is

(19) $$N_{\mathrm{GLR}}=\inf\left\{t>t_{0}:Z_{\mathrm{GLR}}\left(Y_{0,t}\right)\geq c\right\}.$$

The GLR rule in the normal case has been discussed by [33,34]. For the GLR rule in the continuous-time Markov chain, we will study its performance via simulation in Section 4.

### 3.3. An Extended Shiryaev’s Detection Rule

Instead of the non-Bayesian approach, Bayesian rules can be used for sequential surveillance of the rating migration matrix. Ref. [35,36] assumed a geometric prior distribution on *v* and a loss function that takes value *c* for each observation taken at or after *v* and 1 for a false alarm before *v*, provided an optimal sequential detection of the change-time *v* by the Bayesian approach. He showed that an alarm would be triggered as soon as the posterior probability exceeds some fixed level when a change has occurred using optimal stopping theory. By applying Shiryaev’s result to rating transition observations, we have

$$P\left\{v\leq n|Y_{0,n}\right\}=R_{p,n}/\left(R_{p,n}+p^{-1}\right)$$

where *p* is the parameter of the geometric distribution $P\left\{v=n\right\}=p{\left(1-p\right)}^{n-1}$ and $R_{p,n}=,\sum_{k=1}^{n},{\left(1-p\right)}^{-(n-k+1)},L\left({,Y}_{k,n}|\Lambda_{1}\right),/;L\left({,Y}_{k,n}|,\Lambda_{0}\right)$, the Bayes rule declares at time

$$N_{\mathrm{Shi}}=\inf\left\{n\geq 1:R_{p,n}\geq c\right\},$$

when a change has occurred. Following [37,38], we can set p=0 and obtain the Shiryaev–Roberts rule for detecting a change-point in firms’ rating transition dynamics:

$$N_{\mathrm{Shi}.\mathrm{Rob}}=\inf\left\{n\geq 1:\sum_{k=1}^{n}\frac{L(Y_{k,n}|\Lambda_{1})}{L(Y_{k,n}|\Lambda_{0})}\geq c\right\}.$$

According to [37], when p→0, $N_{\mathrm{Shi}.\mathrm{Rob}}$ is asymptotically Bayes-risk efficient.

Ref. [17] extended Shiryaev’s Bayesian detection rule and described asymptotically optimal Bayesian and frequentist solutions to the problem of sequential change-point detection in multiparameter exponential families when the pre- and post-change parameters are unknown. The extended Shiryaev’s rule has the following form:

(20) $$N_{\mathrm{ex}.\mathrm{Shi}}=\inf\left\{n>n_{p}:P\left(v\leq n|v\geq n-k_{p},Y_{0,n}\right)\geq c\right\}.$$

Following [17], we may construct the extended Shiryaev’s Bayesian detection rule for change-points in rating migration matrices as follows. Assume that *v* follows a geometric prior distribution with parameter *p* but is constrained to be lager than $n_{0}$, and $\Lambda_{0},\Lambda_{1}$ are generator matrices in which the ijth elements are independent and identically distributed and follow the gamma prior (5). $\Lambda_{0},\Lambda_{1}$, and *v* are mutually independent. Then

$$P\left(v\leq n|v\geq n-k_{p},Y_{0,n}\right)=\sum_{r=n-k_{p}}^{n}P\left(v=r|Y_{0,n}\right)/\left\{\sum_{r=n-k_{p}}^{n}P\left(v=i|Y_{0,n}\right)+P\left(v>n|Y_{0,n}\right)\right\}.$$

Recall that $f_{0,0}$ and $f_{m,l}$ are defined in (10) and note that, for $n_{0}<r<n$,

$$P\left\{v=r|Y_{0,n}\right\}\propto p{\left(1-p\right)}^{r-1}\frac{f_{1,r-1}f_{r,n}}{f_{0,0}^{2}},\;\;P\left\{v>n|Y_{0,n}\right\}\propto p{\left(1-p\right)}^{n}f_{1,n}/f_{0,0}$$

we obtain the extended Shiryaev’s rule for detecting change-points in generators of continuous-time Markov chains

(21) $$N_{\mathrm{ex}.\mathrm{Shi}}=\inf\left\{n>n_{p}:\sum_{r=n-k_{p}}^{n}\frac{f_{1,r-1}f_{r,n}}{{\left(1-p\right)}^{n-r+1}f_{0,0}f_{1,n}}\geq c\right\}.$$

### 3.4. Bayesian Rules Based on the Stochastic Structural Break Model

The surveillance rules so far assume that there is only one change-point during the sample period, because the quickest detection in most engineering applications assumes that the system can be stopped when a change-point is detected. However, this is not true in many economic and financial applications [39]. When firms’ rating transition dynamics are used as a proxy of the credit market, a structural break in firms’ rating transition dynamics suggests a possible structural break in the credit market. As various kinds of structural breaks might occur in the credit market and one cannot expect that structural breaks will always be detected, surveillance rules that allow misdetection of change-points have the advantage of not disregarding samples that contain no change-points or change-points of small sizes.

To design surveillance rules that allow misdetection of structural breaks in firms’ rating transition dynamics, we consider the SSB model in [8]. Section 2.3 summarizes the SSB model assumption and presents the forward estimates of generator matrices, the probability of the most recent change-points, and the likelihood of the data. We now consider the rule proposed by [18] for a Bayesian change-point model that allows multiple change-points in exponentially distributed families and is based on the location of most recent change-point. We extend the rule of [18] in exponential families to the case of the SSB model. In particular, note that equation (11) provides the probability that the most recent change-point occurs in the period $\left(t_{h-1},t_{l}\right]$; we then construct the surveillance rule as follows:

(22) $$N_{\mathrm{SSB}}=\inf\left\{n>n_{p}:P\left(R_{h}\in \left[t_{h-1},t_{l}\right]|Y_{0,t_{l}}\right)\geq c\right\}=\inf\left\{n>n_{p}:\sum_{h=m}^{l}p_{m,l}\geq c\right\}.$$

## 4. Monte Carlo Results

In this section we go through numerical experiments to discuss the performance of surveillance rules in Section 3. To fix the idea, we assume the rating system has K=4 rating categories in which there is no default category (or absorbing state), and all firms’ rating transitions follow four-state continuous-time Markov chains. For convenience, we further assume that there are always n=1000 firms with their observed ratings at each time *t*.

We first consider the issue of simulating a generator matrix for firms’ rating transitions. There are many ways to simulate generator matrices of continuous-time homogeneous Markov chains. Instead of using estimated generator matrices from real data, we try to simulate a *K*-state generator matrix for rating transitions. We note that firms’ one-year rating transition probability matrix has the following features. First, the diagonal elements in firms’ one-year rating transition probability matrix, or probabilities of staying at the previous rating categories, are usually larger than 0.9. Second, according to CRA’s rating procedures, a firm’s rating is usually upgraded or downgraded sequentially based on the superiority order of rating categories. This suggests that the off-diagonal transition probabilities $p_{ij}$ decrease with the value of |i−j|. Due to these two features, we simulated generator matrix $\Lambda ={\left(\lambda^{\left(i,j\right)}\right)}_{1\leq i,j\leq K}$ in the following way. For 1≤i≠j≤K, we simulated $\lambda^{\left(i,j\right)}$ from a uniform distribution Unif$\left(a^{-|i-j|-1},a^{-|i-j|}\right)$ (*a* is a positive constant), and for 1≤i≤K, we set $\lambda^{\left(i,i\right)}=-\sum_{j\neq i}\lambda^{\left(i,j\right)}$. The positive constant *a* can be chosen to adjust the difference between off-diagonal elements, and in our study, we chose a=16. Table 1 shows three sets of simulated generators and their one-period transition probability matrices. The SVD metrics of three one-period rating transition matrices are given below.

$$M\left(P_{A}\right)=0.07850,\;M\left(P_{B}\right)=0.06716,\;M\left(P_{C}\right)=0.08793.$$

We then carried out the following experiment. Suppose that firm *l*’s (1≤l≤n) rating transitions follow a continuous-time homogeneous Markov chain with generator matrix $\Lambda_{0}$ for t<v and with generator matrix $\Lambda_{1}$ for t≥v, respectively. The following two scenarios are considered.

(S1) $\Lambda_{0}=\Lambda_{A}$ and $\Lambda_{1}=\Lambda_{B}$.
(S2) $\Lambda_{0}=\Lambda_{A}$ and $\Lambda_{1}=\Lambda_{C}$.

Note that in (S1), $|M\left(P_{1}\right)-M\left(P_{0}\right)|=|M\left(P_{A}\right)-M\left(P_{B}\right)|=0.01134$, and in (S2), $|M\left(P_{1}\right)-M\left(P_{0}\right)|=|M\left(P_{A}\right)-M\left(P_{C}\right)|=0.00943$. This indicates that the sizes of changes in transition probability matrices in (S1) and (S2) are very small (For the case $|M\left(P_{1}\right)-M\left(P_{0}\right)|>0.02$, all surveillance rules have no detection delay with the settings of our simulation. Those results are not presented in the paper as all surveillance rules provided very good performances).

We considered four surveillance rules presented in Section 3 and determined the threshold *c* in the surveillance rule as follows. Assume that there is no change in generators of firms’ rating transitions, that is, all rating transitions follow the continuous-time homogeneous Markov chain with generator $\Lambda_{A}$ at each period. We then computed the statistics *Z* in the surveillance rule. We ran said simulation m=1000 times and obtained $\left\{Z_{1},\cdots ,Z_{m}\right\}$. We then chose the 99% quantile of $\left\{Z_{1},\cdots ,Z_{m}\right\}$ as the threshold *c* in the surveillance rule.

For scenarios (S1) and (S2), we considered three locations of structural breaks ν=50,100, and 200; hence, in total we had six scenarios. We ran 500 simulations in each scenario. In each simulation, we generated *n* Markov chains at time t=1,⋯T(T=1000) and applied surveillance rules. Using the 500 detected locations of change-point, we estimated the conditional detection delay (CDD), defined as ${\mathrm{CDD}}_{N}=E\left(N-\nu |N>\nu \right)$, and the false alarm probability (FAP) defined as P(N<ν) when there was a change at ν. Table 2 shows the estimated CDDs and FAPs and their standard errors in parentheses. The estimated FAPs for all surveillance rules increase with the location ν of the change-point. Among the four surveillance rules in the study, the FAP of the LR rule increased fastest and more significantly than the other rules. The FAP of the GLR rule increased less than that of the LR rule, but a little more than the ex.Shi, SSB.prob, and SSB.lr rules. Compared to the FAP, all surveillance rules’ CDDs did not change significantly. In particular, the CDDs of the ex.Shi and SSB rules were larger than those of the LR and GLR rules, and the CDDs of the GLR rule were larger than that of the LR rule. Table 2 seems to suggest the following. Compared with the other three rules, the LR rule had relatively larger FAPs but smaller CDDs. The two Bayesian rules, ex.Shi and SSB, had lower FAPs but sightly larger CDDs than the LR and the GLR rules. The GLR rule seems to have had relatively balanced FAPs and CDDs compared to other rules.

## 5. Data Analysis

In this section we describe an empirical study of using the surveillance rules in Section 4 to analyze structural breaks in the U.S. firms’ rating transition dynamics during 1986–2016 and discuss the implication to structural breaks in the credit market during the period.

### 5.1. Data Description

The data were obtained from COMPUSTAT and consist of Standard and Poor’s monthly credit ratings of firms starting January 1986 and ending February 2017 (The proposed surveillance rules can also be applied to firms’ rating data provided by other credit rating agencies, such as Moody’s and Fitch Group). There were a total of 703,085 rating records and 5802 firms whose ratings were recorded at the end of each month. The raw data contained ten rating categories, AAA, AA, A, BBB, BB, B, CCC, CC, C, and D (default), and 25 rating subcategories which were obtained by adding “+” or “-” to the letter grades of categories and showing relative standing within the major rating categories. For convenience, we grouped C and CC into CCC, as the records in the former two rating categories were few. Hence we had eight rating categories in total, which are denoted by AAA, AA, A, BBB, BB, B, CCC, and D.

**Figure 1:**
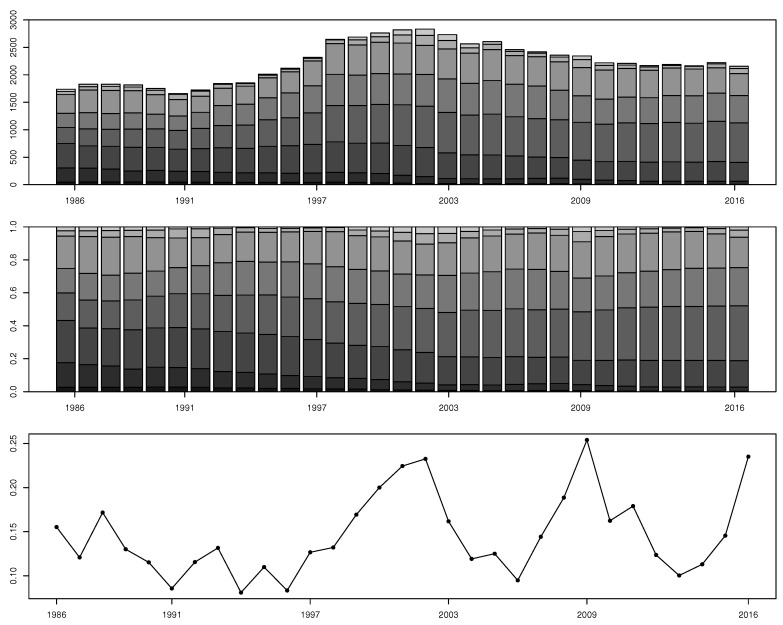
Top panel: The number of firms in each rating category during 1986–2016. Middle panel: The proportion of firms in each rating category during 1986–2016. Bottom panel: The SVD metric of estimated one-year transition probability matrices. (Rating categories AAA, AA, A, BBB, BB, B, CCC, and D are colored from dark gray to light gray or from bottom to top in the top and middle panels).

The top and middle panels of Figure 1 display the number of rated firms and proportion of firms’ ratings in each rating category from 1986 to 2016 and show the following facts. First, the total number of rated firms increased from 1738 in 1986, peaked at 2833 in 2002, and decreased to 2156 in 2016. Second, the numbers and proportions of firms rated AAA, AA, and A decreased significantly during 1986–2016, while the number and proportion of firms rated BBB almost doubled up from 291 to 719 and from 16.7% to 33.3%, respectively, during the whole period. This means the number of firms rated as “investment grade ratings” (i.e., AAA, AA, A, and BBB) increased from 1042 in 1986, peaked at 1455 in 2001, and stayed at the level of 1100s post-2008; in the meanwhile, the proportion of firms with investment grade ratings was above 50% in most years but not in years 2003–2005, 2007, 2009, and 2010. Third, the proportion of firms rated D peaked around 4% during 2002–2003 and at 2.8% at 2009.

As it is not obvious to see whether structural breaks occur in the number and proportion of rated firms, the bottom panel shows the SVD metric of estimated one-year transition probability matrices during 1986–2016. Each year’s transition probability matrix is computed based on a continuous-time homogeneous Markov chain model. The SVD curve ranges from 0.0810 to 0.2540 during the sample period and has three local maximums, which are 0.2326 in 2002, 0.2540 in 2009, and 0.2351 in 2016. The variation of the SVD metric in one-year rating transition probability matrices indicates the non-stationarity of firms’ rating transition dynamics and the instability of the credit market during 1986–2016.

### 5.2. Surveillance of Structural Breaks

Next we carried out surveillance procedures for structural breaks in firms’ rating transition dynamics. Since the pre- and post-change generator matrices of rating transition dynamics are not known, the surveillance procedures include steps of determining threshold values of surveillance rules, computing the surveillance rule statistic, and deciding whether a structural break should be claimed each month, whose details are given below.

Let $t_{0}=$ January 1997, T= February 2017, and *n* be the length of the sliding window in which oserved ratings are used for surveillance. At each month $t=t_{0},t_{0}+1,\cdots ,T$, we used rating data $Y_{t-n+1,t}$ at months {t−n+1,t−n+2,⋯,t} to compute the surveillance statistic and compare it with the corresponding threshold in the surveillance rule. If the statistic exceeded the threshold, a structural break $\tau_{k}=t$ was claimed; otherwise, it was concluded that ratings dynamics in month *t* had no structural break and we proceeded to the next month. Then we obtained a series of structural breaks at $\tau_{1},\tau_{2},\cdots$. For convenience, we also denote $\tau_{0}=t_{0}$.

The threshold *c* in the surveillance rule between two adjacent months of structural breaks $\tau_{k}$ and $\tau_{k+1}$(k=0,1,⋯) is determined by Monte Carlo or bootstrap methods using the rating records $Y_{\tau_{k}-n,\tau_{k}}$ at months $\left\{\tau_{k}-n,\tau_{k}-n+1,\cdots ,\tau_{k}\right\}$. Specifically, for the LR rule (15), we estimate the pre-change generator matrix $\Lambda_{0}$ using data $Y_{\tau_{k}-n,\tau_{k}}$ and then use the estimated generator ${\hat{\Lambda }}_{0}$ to simulate m=1000 groups of rating transitions. Each group of simulated rating transition contains a set of Markov chains in which each Markov chain follows a continuous-time homogeneous Markov chain with generator ${\hat{\Lambda }}_{0}$ and the number of Markov chains is the same as that of firms at period $\left[\tau_{k},\tau_{k}+1\right]$. The LR statistic is computed for each group of simulated rating transitions and the 99% quantile of these *m* LR statistics is chosen as the threshold value $c_{\mathrm{LR}}$ for the LR surveillance rule. For the GLR rule, we used rating records $Y_{\tau_{k}-n,\tau_{k}}$ to generate *m* bootstrap samples and computed the GLR statistic for each bootstrap sample. Then the threshold value $c_{\mathrm{GLR}}$ was chosen as the 99% quantile of the *m* bootstrap GLR statistics. The threshold values $c_{\mathrm{ex}.\mathrm{Shi}}$ and $c_{\mathrm{SSB}}$ were determined similarly to those in the GLR rule via the bootstrap method. Note that all thresholds need to be determined after the month of structural break $\tau_{k}$ is claimed, and the months of structural breaks detected by different surveillance rules are not necessarily the same.

**Figure 2:**
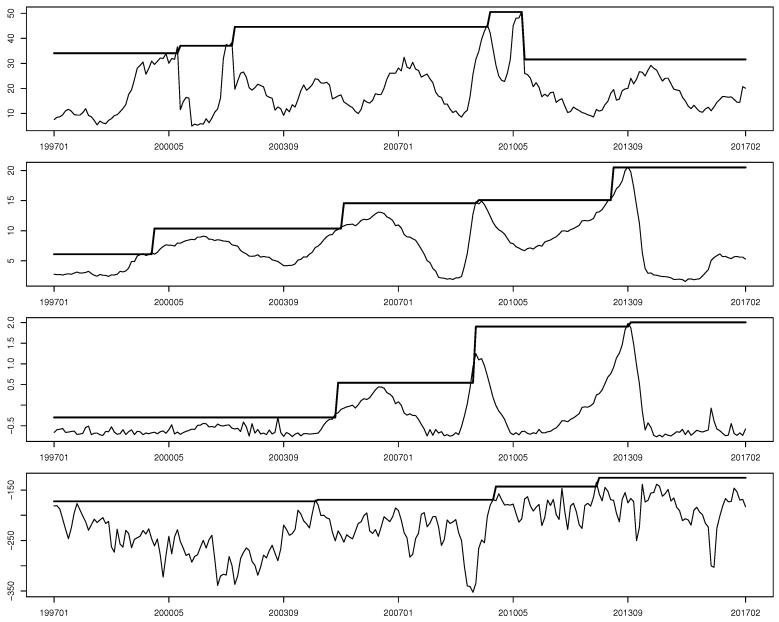
Sequential surveillance of firms’ rating transition dynamics using the LR, GLR, ex.Shi, and SSB rules during January 1997 — February 2017.

We implemented four surveillance rules in the study, which were the LR rule $N_{\mathrm{LR}}$, the GLR rule $N_{\mathrm{GLR}}$, the extended Shiryaev’s rule $N_{\mathrm{ex}.\mathrm{Shi}}$, and the change-point probability rule $N_{\mathrm{SSB}}$ in Section 3 for n=60 months. Figure 2 plots the statistics of four surveillance rules in the moving window from January 1997 to February 2017. The piecewise horizontal lines in each panel of Figure 2 are the thresholds used in each rule for each segmented series. Note that those thresholds were estimated via Monte Carlo or bootstrap methods whenever a structural break was claimed. Besides, depending on the user’s definition of structural breaks, the significance level of those thresholds can also be adjusted in practice. Table 3 lists all structural breaks detected by four surveillance rules. Note that the detected months with structural break are quite different, except that all rules detected changes in 2009, which seems to agree with the fact that the 2007–2008 financial crisis significantly deteriorated the credit market. This is consistent with Figure 2 in which the SVD metric peaks in 2002 and 2009. However, exact months suggested by the four rules have an up to six-month difference, suggesting that some of these rules have relatively large detection delays though the month with the true structural break being unknown. Besides, it seems that the LR and GLR rules are more sensitive to structural changes in generator matrices, as they suggest more structural breaks than other rules.

## 6. Conclusions

This paper studied the problem of the sequential surveillance of structural breaks in firms’ rating transition dynamics. After reviewing some discrete- and continuous-time Markov chain models of firms’ rating transition dynamics, we presented several surveillance rules in statistical process control and applied them to continuous-time Markovian models of rating transition dynamics. Our simulation study showed that the GLR rule performs better than other rules in terms of the trade-off between the false alarm probability and the conditional detection delay. As an example, we applied four surveillance rules to detect structural breaks in the rating transition dynamics of the U.S. firms during 1997–2017. The detected structural breaks show that large structural changes in firms’ rating transitions can be captured, but different surveillance rules tend to provide different structural breaks when they are not significant. The real example also showed that the GLR rule is more sensitive to structural changes in generator matrices.

Besides the specific application of surveillance rules in firms’ rating transition dynamics, this paper shows the application of statistical process control in monitoring financial stability at the regulatory level. Actually, as statistical process control has been discussed and used in various areas of finance, including portfolio optimization, financial time series analysis, and technical analysis of trading, its application to monitoring the stability of the market and the economy does not catch the eye of the statistician or the econometrician. Our study fills in this gap, and the analysis in the paper also suggests that more effective surveillance rules should be studied in subsequent research.

## Figures and Tables

**Table 1:** Simulated generators and their transition probability matrices.

	Λ	P=exp(Λ)
A	$\left(\begin{array}{cccc} -0.08765 & 0.08235 & 0.00481 & 0.00048\\ 0.01711 & -0.08400 & 0.06577 & 0.00111\\ 0.00971 & 0.02892 & -0.09328 & 0.05465\\ 0.00008 & 0.00096 & 0.05006 & -0.05109 \end{array}\right)$	$\left(\begin{array}{cccc} 0.91676 & 0.07569 & 0.00689 & 0.00066\\ 0.01601 & 0.92096 & 0.06032 & 0.00271\\ 0.00911 & 0.02689 & 0.91310 & 0.05091\\ 0.00031 & 0.00158 & 0.04664 & 0.95147 \end{array}\right)$
B	$\left(\begin{array}{cccc} -0.04403 & 0.04189 & 0.00194 & 0.00019\\ 0.03142 & -0.10663 & 0.07277 & 0.00244\\ 0.00294 & 0.04328 & -0.09949 & 0.05327\\ 0.00003 & 0.00197 & 0.02572 & -0.02772 \end{array}\right)$	$\left(\begin{array}{cccc} 0.95755 & 0.03893 & 0.00322 & 0.00031\\ 0.02926 & 0.90089 & 0.06577 & 0.00408\\ 0.00337 & 0.03918 & 0.90736 & 0.05008\\ 0.00010 & 0.00236 & 0.02422 & 0.97331 \end{array}\right)$
C	$\left(\begin{array}{cccc} -0.09537 & 0.09434 & 0.00093 & 0.00010\\ 0.05179 & -0.11318 & 0.06094 & 0.00045\\ 0.00287 & 0.01145 & -0.09888 & 0.08456\\ 0.00007 & 0.00108 & 0.05023 & -0.05138 \end{array}\right)$	$\left(\begin{array}{cccc} 0.91125 & 0.08509 & 0.00345 & 0.00022\\ 0.04679 & 0.89550 & 0.05493 & 0.00278\\ 0.00288 & 0.01048 & 0.90812 & 0.07852\\ 0.00016 & 0.00127 & 0.04667 & 0.95191 \end{array}\right)$

**Table 2:** False alarm probability and conditional detection delay.

	Rules	FAP			CDD		
		ν=50	ν=100	ν=200	ν=50	ν=100	ν=200
(S1)	LR	0.248 (0.019)	0.354 (0.021)	0.810 (0.017)	2.420 (0.823)	1.659 (0.130)	1.916 (0.269)
	GLR	0.062 (0.011)	0.120 (0.014)	0.342 (0.021)	2.746 (0.041)	2.607 (0.041)	2.596 (0.045)
	ex.Shi	0.036 (0.008)	0.076 (0.012)	0.260 (0.019)	4.340 (0.052)	4.284 (0.051)	4.300 (0.056)
	SSB	0.036 (0.008)	0.064 (0.011)	0.238 (0.019)	4.315 (0.053)	3.667 (0.049)	3.766 (0.052)
(S2)	LR	0.278 (0.020)	0.342 (0.021)	0.782 (0.018)	0.343 (0.040)	0.571 (0.062)	0.358 (0.063)
	GLR	0.064 (0.011)	0.120 (0.015)	0.376 (0.022)	2.068 (0.033)	1.932 (0.036)	1.987 (0.040)
	ex.Shi	0.070 (0.011)	0.092 (0.013)	0.274 (0.020)	3.357 (0.041)	3.236 (0.043)	3.154 (0.046)
	SSB	0.064 (0.011)	0.074 (0.012)	0.248 (0.019)	3.340 (0.041)	2.659 (0.041)	2.697 (0.043)

**Table 3:** Detected structural breaks in firms’ rating transition dynamics.

Rules	Structural Breaks
LR	August 2000, March 2002, August 2009, August 2010
GLR	November 1999, May 2005, September 2005, June 2009, March 2013
ex.Shi	July 2003, March 2005, March 2009, September 2013
SSB	August 2004, October 2009, October 2012

## Abbreviations

The following abbreviations are used in this manuscript:

CUSUM	cumulative sum
GLR	Generalized likelihood ratio
SSB	Stochastic structural break
SVD	Singular value decomposition
LR	Likelihood ratio
